# Unhealthy Lifestyle Behaviours and Psychological Distress: A Longitudinal Study of Australian Adults Aged 45 Years and Older

**DOI:** 10.3390/ijerph19074399

**Published:** 2022-04-06

**Authors:** Emma S. George, Ian Davidson, Aymen El Masri, Tanya Meade, Gregory S. Kolt

**Affiliations:** 1 School of Health Sciences, Western Sydney University, Penrith, NSW 2751, Australia; a.elmasri@westernsydney.edu.au (A.E.M.); g.kolt@westernsydney.edu.au (G.S.K.); 2 Translational Health Research Institute, Western Sydney University, Penrith, NSW 2751, Australia; t.meade@westernsydney.edu.au; 3 School of Psychology, Western Sydney University, Penrith, NSW 2751, Australia; ian.davidson878@gmail.com

**Keywords:** psychological distress, longitudinal study, lifestyle behaviours, mental health

## Abstract

Individual associations between lifestyle behaviours and mental health have been established; however, evidence on the clustering of these behaviours and the subsequent impact on mental health is limited. The purpose of this study was to examine cross-sectional and longitudinal associations between combined unhealthy lifestyle behaviours (physical activity, sitting time, sleep duration, processed meat consumption, vegetable consumption, fruit consumption, smoking status, alcohol consumption) and the development of psychological distress (measured using the Kessler Psychological Distress Scale) in a large Australian sample. Participants were 163,707 Australian adults from the 45 and Up Study. Data from baseline (2006–2009) and follow-up wave 1 (2012) were analysed using binary logistic regression. The odds of reporting high or very high psychological distress at follow-up were significantly higher for those reporting five (AOR = 2.36; 95% CI 1.41–3.97, *p* = 0.001) or six or more (AOR = 3.04; 95% CI 1.62–5.69, *p* = 0.001) unhealthy lifestyle behaviours, in comparison to those reporting no unhealthy lifestyle behaviours at baseline. These findings suggest that a holistic, multi-faceted lifestyle approach addressing multiple behaviours may be required to support and promote positive mental health and to reduce the likelihood of psychological distress.

## 1. Introduction

Mental disorders such as anxiety and depression are major contributors to the global burden of disease [1] and have a debilitating impact on the individual, family and friends, and the broader community [2]. From an economic viewpoint, in Australia, AUD 9.9 billion was spent on mental health services in 2017–2018, which equated to 7.6% of government health expenditure [3]. Common treatments for disorders such as depression and anxiety often involve psychotherapy and/or pharmacotherapy. Although these treatments have been shown to be efficacious in reducing symptomology [4,5], it is important to consider the role of nonpharmacological approaches, such as the promotion of healthy lifestyle behaviours, in the prevention and management of mental disorders.

By way of example, physical activity participation is associated with a reduced likelihood of depression [6], and available evidence suggests that sedentary behaviour may be associated with increased depression risk [7]. Recent systematic reviews have established dietary intake as an important consideration for mental health [8,9] and evidence suggests that high volumes of alcohol consumption [10], smoking [11], and sub-optimal sleep durations [12] are also associated with increased risk of depression and should be considered for the prevention of mental disorders.

Despite well-established evidence on the mental health benefits of these lifestyle behaviours, a large proportion of adults fail to meet recommended guidelines related to these behaviours. In Australia in 2017–2018, for example, only 45% of adults aged 18 years and over were sufficiently active, while only 4% of men and 11% of women were consuming the recommended number of daily servings for vegetables, and 47% of men and 56% of women were meeting recommendations for fruit intake [13]. Data from 2019 showed that approximately 16% of Australian adults aged in their 40s and 50s reported smoking daily, and adults aged from 40 to 59 years were more likely to drink at levels exceeding lifetime risk guidelines [13].

In recent years, there has been increasing interest in the association between multiple lifestyle behaviours and mental health outcomes. Much of this evidence has focused on four major risk factors for poor health—physical inactivity, alcohol consumption, poor diet, and smoking [14]; however, emerging evidence suggests that sleep and sedentary behaviour may also be important. Sarris and colleagues recently examined the longitudinal associations between six lifestyle factors (physical activity, healthy diet, sleep, screen time, tobacco smoking, and alcohol consumption) and depressed mood in a sample of 84,860 participants from the UK Biobank [15]. Results showed that screen time and sleep were prospectively associated with depressed mood in both participant groups. In those without depressive disorders at baseline, a healthy baseline diet was also significantly associated with a decreased frequency of depressed moods.

The focus on multiple health behaviours is important in this line of research, as unhealthy behaviours are frequently interrelated and often occur in clusters. Further, when higher numbers of these unhealthy behaviours are found in combination, the likelihood of poor health outcomes increases [16]. Associations between behavioural risk factors and disease outcomes are also multi-faceted [14], and understanding the cumulative effect of lifestyle behaviours on health outcomes can inform the development of health policy and promotion approaches more effectively than examining individual lifestyle behaviours in isolation [17].

Several studies have examined the cross-sectional relationship between indices of healthy or unhealthy lifestyle behaviours on mental health outcomes [18,19,20,21]. In a sample of 10,364 adults aged 18 years and over, Harrington et al. [22] found that participants meeting recommendations for four protective lifestyle behaviours (physical activity, fruit and vegetable consumption, being a non-smoker, and moderate alcohol consumption) were significantly more likely to have better mental health than those who were not meeting recommendations. Loprinzi and Mahoney [19] found a dose–response relationship between the concurrent occurrence of healthy lifestyle behaviours (physical activity, healthy eating, and being a non-smoker) and depression. Similarly, Velten et al. [21] found that higher combined unhealthy lifestyle scores were associated with higher rates of poor mental health outcomes such as anxiety and depression, and in a longitudinal study, higher healthy lifestyle scores were associated with a reduced risk of depression at follow-up [23]. At present, there are no longitudinal studies in an Australian context examining the combined influence of unhealthy lifestyle behaviours on mental health outcomes.

The aim of this study was to investigate both the cross-sectional and longitudinal associations between combined lifestyle behaviours and the development of psychological distress in a large Australian sample. It is hypothesised that the higher the unhealthy lifestyle index score, the higher the odds of developing psychological distress.

## 2. Materials and Methods

### 2.1. Data

Data for this study were drawn from the 45 and Up Study [24]. The 45 and Up Study comprises 267,153 randomly selected adults aged 45 years and over (18% response rate) from New South Wales, the most populous state in Australia. Although sampling was random, participants over 80 years of age and participants living in remote areas were oversampled. The 45 and Up Study is the largest study in the Southern Hemisphere investigating healthy aging. Baseline data were collected in a series of waves between 2006 and 2009. Eligible participants completed a mailed-out gender-specific questionnaire (found at: https://www.saxinstitute.org.au/our-work/45-up-study/questionnaires, accessed on 21 July 2021) and provided written consent, including consent for follow-up. Questionnaires were sent back by prepaid post. The first follow-up wave was mailed out to the first wave of baseline participants (*n* = 41,440) in September 2012 and had approximately 27,000 respondents. The 45 and Up Study was approved by the University of New South Wales Human Ethics Committee. Reciprocal ethics approval for the current study was obtained from the Western Sydney University Human Research Ethics Committee (H10930).

### 2.2. Participants

Participants were a subsample drawn from the 45 and Up Study with complete data on all variables at baseline (*n* = 163,707) and follow-up (*n* = 16,502).

### 2.3. Study Variables

Lifestyle behaviour variables used to create the unhealthy lifestyle index were physical activity, sitting time, sleep duration, processed meat consumption, vegetable consumption, fruit consumption, smoking status, and alcohol consumption. These behaviours were selected for inclusion in the index due to their association with poorer mental health outcomes [6,7,8,9,10,11,12,14,15].

#### 2.3.1. Physical Activity

Physical activity was assessed using the Active Australia Survey [25]. The survey has been used across several Australian populations and has acceptable test–retest reliability [26]. The survey includes questions regarding frequency and the amount of time that participants engage in walking and moderate and vigorous intensity physical activity. Time spent in physical activity each week was calculated. Participants’ responses were coded as 0 if they reported ≥150 min of total physical activity per week. Participants were coded as 1 if they did not meet this level of physical activity, in accordance with Australian Government physical activity guidelines [27].

#### 2.3.2. Sitting Time

Sitting time was defined as the number of hours spent sitting each day. Participants responded to the question “About how many hours in each 24-h day do you usually spend doing the following?”, which included sitting, sleeping, watching television or using a computer, and standing. For sitting time, participants’ responses were coded 1 if they reported sitting for >7 h each day and 0 if they reported sitting for ≤7 h each day, which is in accordance with a meta-analysis demonstrating the increased risk of all-cause mortality for sitting >7 h per day [28].

#### 2.3.3. Sleep Duration

Sleep duration was assessed using the question “About how many hours in each 24-h day do you usually spend doing the following?”, with participants asked to report time spent sleeping at night and during naps. Participant responses were coded 1 if they reported sleep durations of <7 and >9 h per day and coded 0 if they reported 7–9 h per day, which is in accordance with a meta-analysis demonstrating the link between shorter and longer sleep durations with cardiovascular outcomes [29].

#### 2.3.4. Processed Meat Consumption

Participants responded to the question “About how many times each week do you eat processed meat?” Participant responses were coded as 1 if they reported any processed meat consumption in the last week and 0 if they reported no consumption of processed meat in the last week. This is in line with evidence relating to the harmful effects of processed meat consumption [30].

#### 2.3.5. Vegetable Consumption

A participant’s response to the question “About how many serves of vegetables do you usually eat each day?” was used to assess vegetable consumption. In accordance with Australian dietary guidelines [31], participant responses were coded as 0 if they reported ≥5 servings of vegetables daily and 1 if they reported <5 servings daily.

#### 2.3.6. Fruit Consumption

Fruit consumption was assessed using the question “About how many serves of fruit do you have each day?” In accordance with Australian dietary guidelines [31], participant responses were coded as 0 if they reported ≥2 servings of fruit daily and 1 if they reported <2 servings of fruit daily.

#### 2.3.7. Tobacco Smoking

To assess smoking status, participants responded to the questions “Have you ever been a regular smoker” and “Are you a regular smoker now?” Current smoking status was coded as 1 if participants reported being a current smoker. A code of 0 was given if participants were non-smokers (those who have never smoked and ex-smokers).

#### 2.3.8. Alcohol Consumption

Information on weekly alcohol consumption was requested. Participants responded to the question “About how many alcoholic drinks do you have each week?” Following recommended guidelines and previous studies [32], participant responses were coded as 1 if they reported consuming >14 standard drinks per week and 0 if they reported consuming ≤14 standard drinks per week.

#### 2.3.9. Unhealthy Lifestyle Index

The 8 lifestyle behaviours (physical activity, sitting time, sleep duration, processed meat consumption, vegetable consumption, fruit consumption, tobacco smoking, and alcohol consumption) were summed to give an unhealthy lifestyle score that ranged from 0 to 8. This approach is similar to that used in previous studies [21,22,33,34]. Due to low cell counts toward the upper end of the lifestyle index scores, ≥6 unhealthy lifestyle behaviours were combined. Higher scores on this index indicate a higher number of unhealthy lifestyle behaviours.

#### 2.3.10. Outcome Variable: Psychological Distress

The outcome variable of psychological distress was measured with the Kessler Psychological Distress Scale (K10), a 10-item questionnaire that measures psychological distress by examining depressive (5 items) and anxious (5 items) symptomology [35,36]. Participants were required to answer questions on a 5-point scale, with scores ranging from 1 (none of the time) to 5 (all of the time). Final scores on the K10 range from 10 to 50. High to very high psychological distress was defined as having a K10 score ≥ 22 [35,37]. In line with several other studies [38,39,40], the K10 variable was dichotomised using recommended cut-points for low to moderate and high to very high psychological distress [35] to explore the development of high or very high psychological distress at follow-up. The K10 has been extensively used in research and clinical settings and is considered to have strong reliability and validity (Cronbach’s alpha 0.93) [36], including in older adult populations [41].

### 2.4. Statistical Analyses

Descriptive statistics, including frequencies, were used to examine sociodemographic characteristics, lifestyle behaviours, and the unhealthy lifestyle index. Using baseline data, logistic regression was conducted to examine the relationship between individual lifestyle behaviours (physical activity, sitting time, sleep duration, processed meat consumption, vegetable consumption, fruit consumption, smoking status, and alcohol consumption) and psychological distress. Logistic regression analysis was then conducted using psychological distress at follow-up as the primary outcome variable to evaluate whether baseline unhealthy lifestyle index scores were predictive of incidence of high/very high psychological distress. Those with existing high/very high psychological distress at baseline were excluded from the longitudinal analysis, given that the focus was on the development of psychological distress over time. In line with previous studies, a reference category of zero was used to compare those reporting no unhealthy lifestyle behaviours to those reporting one or more [14,33]. All analyses were adjusted for possible confounding influence by adjusting for age, gender, marital status, education, income, employment status, and Body Mass Index (BMI). Unadjusted odds ratios (OR) and adjusted odds ratios (AOR), with 95% confidence intervals (95% CI), are shown for the logistic regression models. *p*-values of *p* < 0.05 were interpreted as statistically significant. Statistical analyses were conducted using SPSS statistics 23. In line with approaches used in previous studies using the 45 and Up Study cohort [33,42], exclusions were made for those with an incomplete lifestyle index (missing ≥ 1 lifestyle behaviour), incomplete K10 data (missing ≥ 1 response), and other independent variables.

## 3. Results

### 3.1. Cross-Sectional Results

Table 1 presents demographic information for the sample. Of the 163,707 participants with valid K10 data at baseline, the mean baseline age of participants was 60.73 years (SD = 10.30). In total, 93.1% of participants reported low to moderate levels of psychological distress, while the remaining 6.9% of participants reported high to very high levels of psychological distress.

Table 2 presents the number and percentages for each lifestyle behaviour dichotomised into two categories: meeting and not meeting recommendations. Of the eight indicators, processed meat consumption was the highest (75.3%) reported instance of not meeting recommendations. Also presented are the unhealthy lifestyle index score, the highest being for two (26%) and three (27%) unhealthy indicators.

Table 3 provides baseline results for each lifestyle behaviour and the unhealthy lifestyle index, and their relationship to psychological distress using unadjusted and adjusted odds ratios. Processed meat (AOR = 1.01; 95% CI 0.97–1.06, *p* = 0.56) and high alcohol consumption (AOR = 0.97; 95% CI 0.92–1.03, *p* = 0.32) were the only individual lifestyle behaviours not significantly associated with psychological distress. Each other lifestyle behaviour including insufficient physical activity (AOR = 1.81; 95% CI 1.74–1.89, *p* < 0.001), high sitting time (AOR = 1.37; 95% CI 1.31–1.43, *p* < 0.001), sub-optimal sleep duration (AOR = 2.37; 95% CI 2.28–2.47, *p* < 0.001), insufficient vegetable consumption (AOR = 1.16; 95% CI 1.11–1.21, *p* < 0.001), insufficient fruit consumption (AOR = 1.29; 95% CI 1.24–1.34, *p* < 0.001), and current smoking status (AOR = 1.83; 95% CI 1.73–1.95, *p* < 0.001) were significantly associated with psychological distress. In comparison to the reference category (no unhealthy behaviours), higher odds of psychological distress were observed among those who reported two or more unhealthy behaviours.

### 3.2. Longitudinal Results

After excluding participants reporting high/very high psychological distress at baseline (to examine the development of psychological distress), there were 16,502 participants at follow-up. Of these, 96.2% of participants reported low to moderate levels of psychological distress, while the remaining 3.8% of participants reported high to very high levels of psychological distress.

Table 4 presents the unadjusted and adjusted odds ratios for developing psychological distress at follow-up from the baseline unhealthy lifestyle index. Participants with five (AOR = 2.36; 95% CI 1.41–3.97, *p* = 0.001) and six or more (AOR = 3.04; 95% CI 1.62–5.69, *p* = 0.001) unhealthy lifestyle behaviours at baseline had significantly increased odds of developing psychological distress at follow-up compared to those with no unhealthy lifestyle behaviours.

## 4. Discussion

These findings from a large Australian cohort study show that participants with low or moderate psychological distress and a higher score on the unhealthy lifestyle index at baseline were more likely to report high or very high psychological distress at follow-up in comparison to those who had a lower score on the unhealthy lifestyle index at baseline. The association between cumulative unhealthy lifestyle behaviours and psychological distress appeared stronger at baseline, with modest increases in odds ratios observed for lifestyle index scores of two or more. At follow-up, the odds of psychological distress increased linearly with increasing scores on the unhealthy lifestyle index; however, significant associations were only observed in index scores of five or more, where unhealthy lifestyle behaviours are highly cumulative.

This differs from previously reported findings, such as those from Adjibade et al. [23], which found associations between lifestyle behaviours and depressive symptoms when as few as three behaviours were clustered. In a cohort of French adults aged 18 years and over, Adjibade et al. [23] found a one-point increase in a healthy lifestyle index score (comprising dietary intake, weight, physical activity, smoking, and alcohol consumption) resulted in a 10% decrease in the risk of depressive symptoms approximately five years post-baseline. In comparison to those reporting zero to two healthy lifestyle behaviours, those reporting three, four, and five healthy lifestyle behaviours had a 16%, 26%, and 25% reduction in risk of depressive symptoms at follow-up, respectively, highlighting the benefits of cumulative healthy lifestyle behaviours and mental health.

Cross-sectional analyses of each individual unhealthy lifestyle behaviour, at baseline, showed that insufficient physical activity, lower than recommended fruit and vegetable consumption, smoking, high levels of sitting, and sub-optimal sleep were all associated with higher odds of psychological distress. These findings are consistent with other cross-sectional findings [15,19] and highlight the importance of healthy lifestyles for mental health. Interestingly, alcohol consumption and processed meat intake were not significantly associated with psychological distress at baseline. Sarris et al. [15] reported that a higher frequency of alcohol consumption was associated with lower odds of depressed mood in individuals with major depressive disorder, positing that alcohol may in fact be used as a form of self-medication.

Cross-sectional results of the current study also showed that higher scores on the unhealthy lifestyle index were associated with increased odds of psychological distress at baseline, even after adjusting for potential confounders including age, gender, marital status, education, income, employment status, and BMI. The odds of reporting high or very high psychological distress at baseline were five times higher in those reporting six or more unhealthy lifestyle behaviours compared with those reporting no unhealthy behaviours.

In relation to prevention and treatment, mental health clinicians are often trained to, and may often largely focus on, the assessment and treatment of mental disorders and their associated symptoms [43] rather than the potential underlying causes. This can lead to an underestimation of the importance of lifestyle factors in the development and maintenance of psychological illness [43]. The present findings, as well as those of other recent studies [15,23,44], suggest that prevention and reduction of psychological distress may also be achieved by focusing on the lifestyle behaviours of the individual. For example, a recent randomised controlled trial testing the efficacy of a dietary improvement intervention for the treatment of major depressive episodes [44] resulted in significant reductions (32% vs. 8% in control group) in symptoms of depression. The prevention of mental illness or the management before acute symptoms present or reoccur is easier to manage and more cost-effective than when the illness is in its developed state [45]. Hence, a multi-faceted approach, targeting multiple lifestyle behaviours for the prevention of psychological distress, may be beneficial.

There are several potential limitations that must be considered when interpreting the results from this study. The use of data from the 45 and Up Study limits the generalisation of findings to younger people; however, as chronic diseases increase and healthy lifestyle behaviours such as physical activity tend to decrease with age [13], this is an important age group for the promotion of health. Furthermore, the use of self-report data and the absence of objective information may mean that participants respond in a more socially desirable manner; this may be particularly so for certain lifestyle behaviours such as physical activity [46]. Despite the recognised psychometric properties of the Active Australia Survey, it has been previously reported that this particular measurement tool tends to overestimate physical activity levels in comparison to more comprehensive tools such as the Behavioral Risk Factor Surveillance System [47]. This may partially explain the reason for the high levels of physical activity reported in the study sample (81%) in comparison to the general Australian population aged 18 years and over (45%). Participant self-selection may also impact the overall result with an over-representation of those who are more engaged with healthy lifestyle behaviours [48]. Sitting time has been established as an independent risk factor for chronic disease, and prolonged periods of sitting have been shown to be deleterious, even in those achieving recommended levels of physical activity [49]. While it is possible that individuals reporting ≥150 min of physical activity were also reporting >7 h per day of sitting time, both variables were included in the unhealthy lifestyle index due to the independent nature of risks associated with each behaviour.

To create an overall unhealthy lifestyle index, participant responses for the eight selected lifestyle behaviours were dichotomised to create a “healthy” and “unhealthy” category. It should be acknowledged that this dichotomisation may lead to a reduction in sensitivity; however, the use of a combined lifestyle index means behaviours, which are likely to cluster [16,17], are considered holistically and not in isolation. Lifestyle indices, such as the one created in the current study, have been used to examine the relationship between unhealthy lifestyles and socioeconomic status [34], all-cause mortality [14], and clustering of unhealthy lifestyles [33], to provide evidence on the important cumulative effect of these behaviours on health outcomes of interest.

A major strength of this study is its longitudinal design and a larger sample size than many previous studies in the area. Longitudinal research can provide important insights into the temporal sequence of events and can also help to address potential uncertainty around reverse causality [50,51]. Despite these strengths, it is also important to note that only 3.8% of the sample at follow-up had high or very high psychological distress, and we cannot be certain that baseline unhealthy lifestyle behaviours were the direct cause of the development of psychological distress at follow-up. Future longitudinal research will likely help elucidate and add further critical information regarding any causal roles between lifestyle behaviours and mental ill health, providing greater opportunity to explore potential dose-response relationships between individual and combined lifestyle behaviours and psychological distress. Although the overall response rate for the 45 and Up Study was 18%, estimates from the 45 and Up Study are consistent with other population-based studies, including the NSW Population Health Survey—a computer-assisted telephone interview with a response rate of approximately 60% [48]. Nevertheless, inferences drawn from this study must be interpreted conservatively.

## 5. Conclusions

This study builds on existing evidence by creating a comprehensive unhealthy lifestyle index comprising emerging risk factors such as sitting time and sub-optimal sleep. Using an unhealthy lifestyle index, the current study found that cumulative baseline unhealthy behaviours were significantly associated with increased odds of reporting psychological distress at follow-up. These findings suggest that a holistic, multi-faceted lifestyle approach may be required to support and promote positive mental health and to reduce the likelihood of psychological distress. Future research could focus on multiple waves of follow-up with longer durations, wider age ranges, stratification of results by sex, alternate measurement tools, and opportunities for data linkage.

## Figures and Tables

**Table 1:** Demographic characteristics of the sample at baseline.

Demographics	*n*	%
Age		
45–54	56,199	34.3%
55–64	56,738	34.7%
65–74	32,138	19.6%
≥75	18,632	11.4%
Gender		
Male	78,942	48.2%
Female	84,765	51.8%
Marital Status		
Single	36,268	22.2%
In a relationship	127,439	77.8%
Education		
No education	14,413	8.8%
School certificate	33,030	20.2%
Higher school certificate	16,409	10.0%
Diploma/Apprenticeship	54,777	33.5%
University	45,078	27.5%
Employment status		
Unemployed	75,909	46.4%
Employed	87,798	53.6%
Income		
≤AUD 19,999	28,047	17.1%
AUD 20,000–49,999	42,752	26.1%
AUD 50,000–69,999	19,869	12.1%
≥AUD 70,000	48,023	29.3%
Prefer not to answer	25,016	15.3%
BMI categories ^		
Underweight	59,724	36.5%
Healthy	1860	1.1%
Overweight	65,548	40.0%
Obese	36,575	22.3%

Note. Total number of participants was 163,707. ^ World Health Organization BMI categories.

**Table 2:** Lifestyle behaviours and unhealthy lifestyle index at baseline.

Lifestyle Behaviours and Unhealthy Lifestyle Index	*n*	%
Physical activity		
≥150 min per week	132,575	81.0%
<150 min per week	31,132	19.0%
Sitting time		
≤7 h per day	120,371	73.5%
>7 h per day	43,336	26.5%
Sleep duration		
7–9 h per day	128,727	78.6%
<7 or >9 h per day	34,980	21.4%
Processed meat consumption		
None per week	40,394	24.7%
At least once per week	123,313	75.3%
Vegetable consumption		
≥5 servings per day	51,032	31.2%
<5 servings per day	112,675	68.8%
Fruit consumption		
≥2 servings per day	95,843	58.5%
<2 servings per day	67,864	41.5%
Alcohol consumption		
≤14 standard drinks per week	138,697	84.7%
>14 standard drinks per week	25,010	15.3%
Smoking status		
Non-smoker	152,427	93.1%
Current smoker	11,280	6.9%
Unhealthy lifestyle index		
Zero	5948	3.6%
One	24,535	15.0%
Two	42,392	25.9%
Three	44,466	27.2%
Four	29,596	18.1%
Five	12,738	7.8%
≥Six	4032	2.5%

Note. Total number of participants was 163,707. The number on the unhealthy lifestyle index relates to the number of unhealthy behaviours.

**Table 3:** Baseline odds of psychological distress with lifestyle behaviours and unhealthy lifestyle index using unadjusted odds ratios (OR) and adjusted odds ratios (AOR).

Lifestyle Behaviour	OR	CI [95%]	AOR	CI [95%]
Physical activity				
≥150 min per week	1.00 [Ref]		1.00 [Ref]	
<150 min per week	1.98 ***	1.90–2.07	1.81 ***	1.74–1.89
Sitting time				
≤7 h per day	1.00 [Ref]		1.00 [Ref]	
>7 h per day	1.25 ***	1.20–1.30	1.37 ***	1.31–1.43
Sleep duration				
7–9 h per day	1.00 [Ref]		1.00 [Ref]	
<7 or >9 h per day	2.73 ***	2.62–2.84	2.37 ***	2.28–2.47
Processed meat consumption				
None per week	1.00 [Ref]		1.00 [Ref]	
At least once per week	1.01	0.97–1.06	1.01.	0.97–1.06
Vegetable consumption				
≥5 servings per day	1.00 [Ref]		1.00 [Ref]	
<5 servings per day	1.16 ***	1.11–1.21	1.16 ***	1.11–1.21
Fruit consumption				
≥2 servings per day	1.00 [Ref]		1.00 [Ref]	
<2 servings per day	1.42 ***	1.36–1.47	1.29 ***	1.24–1.34
Alcohol consumption				
≤14 standard drinks per week	1.00 [Ref]		1.00 [Ref]	
>14 standard drinks per week	0.89 ***	0.84–0.94	0.97	0.92–1.03
Smoking status				
Non-smoker	1.00 [Ref]		1.00 [Ref]	
Current smoker	2.85 ***	2.70–3.01	1.83 ***	1.73–1.95
Unhealthy lifestyle index				
Zero	1.00 [Ref]		1.00 [Ref]	
One	1.12	0.96–1.29	1.15	0.99–1.33
Two	1.43 ***	1.25–1.65	1.50 ***	1.30–1.73
Three	1.82 ***	1.58–2.09	1.90 ***	1.65–2.19
Four	2.42 ***	2.10–2.78	2.45 ***	2.13–2.83
Five	3.64 ***	3.16–4.20	3.51 ***	3.03–4.07
≥Six	5.97 ***	5.12–6.98	5.14 ***	4.38–6.04

Note. ** *p* < 0.01, *** *p* < 0.001; Adjusted for age, gender, marital status, education, income, employment status, and BMI; CI—confidence interval.

**Table 4:** Odds of developing psychological distress at follow-up with increases in the baseline unhealthy lifestyle index using unadjusted odds ratios (OR) and adjusted odds ratios (AOR).

Unhealthy Lifestyle Index	OR	CI [95%]	AOR	CI [95%]
Zero	1.00 [Ref]		1.00 [Ref]	
One	0.88	0.54–1.46	0.89	0.53–1.47
Two	1.08	0.67–1.74	1.10	0.68–1.77
Three	1.36	0.85–2.18	1.43	0.89–2.31
Four	1.47	0.91–2.37	1.51	0.92–2.46
Five	2.32 **	1.40–3.85	2.36 **	1.41–3.97
≥Six	3.06 ***	1.66–5.64	3.04 **	1.62–5.69

Note. ** *p* < 0.01, *** *p* < 0.001; Adjusted for age, gender, marital status, education, income, employment status, and BMI; CI—confidence interval.

## Data Availability

The data that support the findings of this study are available from the Sax Institute, but restrictions apply to the availability of these data, which were used under license for the current study so are not publicly available. The dataset used in the analysis of this study is available upon reasonable request and with the permission of the Sax Institute.
